# Associations between heart rate asymmetry expression and asymmetric detrended fluctuation analysis results

**DOI:** 10.1007/s11517-022-02645-6

**Published:** 2022-08-24

**Authors:** J. Piskorski, M. Kośmider, D. Mieszkowski, S. Żurek, B. Biczuk, S. Jurga, T. Krauze, A. Wykrętowicz, P. Guzik

**Affiliations:** 1 Institute of Physics, University of Zielona Góra, Zielona Góra, Poland; 2 Faculty of Medicine and Health Sciences, University of Zielona Góra, Zielona Góra, Poland; 3 Department of Cardiology – Intensive Therapy, Poznań University of Medical Sciences, Poznań, Poland

**Keywords:** HRV, HRA, ADFA, Asymmetry, RR intervals

## Abstract

**Abstract:**

The relation between recently established asymmetry in Asymmetric Detrended Fluctuation Analysis (ADFA) and Heart Rate Asymmetry is studied. It is found that the ADFA asymmetric exponents are related both to the overall variability and to its asymmetric components at all studied time scales. We find that the asymmetry in scaling exponents, i.e., $\alpha ^{+}<\alpha ^{-}$ is associated with both variance-based and runs-based types of asymmetry. This observation suggests that the physiological mechanisms of both types are similar, even though their origins and mathematical methods are very different.

**Graphical abstract:**

The graphical abstract demonstrates strong, nonlinear association between the expression of Heart Rate Asymmetry measured using relative descriptors and the Asymmetric Detrended Fluctuation Analysis results. It is clear that there is a strong relation between the two theoretically disparate approaches to signal analysis. The technique to demonstrate the association is *loess* fit. 
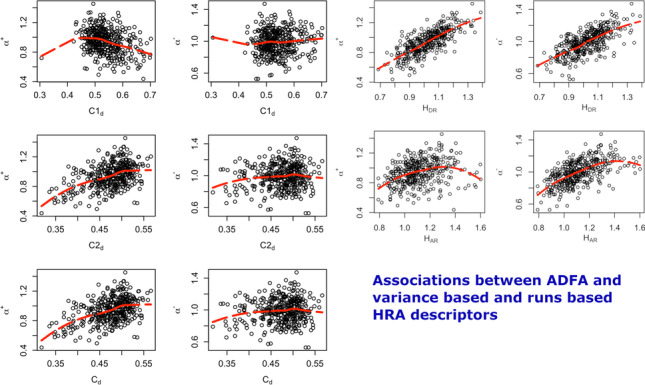

## Introduction

Asymmetry in the $RR$ intervals time series has recently found interest in the heart rate variability research [1–5]. Starting from the most generic approaches to time-irreversibility, which in fact spans an enormous number of physiological phenomena and heart rate variability (HRV) measures [5–7] through specialized predictive methods like Phase Rectified Signal Averaging (PRSA) [8, 9] or Heart Rate Turbulence (HRT) [10–12] down to phenomena like Heart Rate Asymmetry (HRA) along with its mathematical tooling.

A recent addition to the literature is the Asymmetric Detrended Fluctuation Analysis, which was developed mainly for studying asymmetric correlations in economic time series [13, 14], but when applied to the $RR$ intervals time series, revealed an asymmetric physiological effect [15, 16].

HRA includes asymmetric effects defined and established in the variance of the $RR$ intervals time series, in its structure and complexity. In all of these areas asymmetry is prevalent, consistent and unidirectional [1, 2, 17–20]. Thus, we hypothesize that HRA and the above-mentioned ADFA results should be connected, and our aim in this paper is to relate the $\alpha ^{+}<\alpha ^{-}$ asymmetric effect established in ADFA to HRA.

### Asymmetric detrended fluctuation analysis

Detrended fluctuation analysis is one of the most often used methods for analyzing the time series of $RR$ intervals. The main information it provides is the scaling properties of noise left over after detrending the time series. The details of the method may be found in [21–23], and they will be reviewed below very quickly so as to establish the notation.

Let us define the $RR$ intervals time series as the distance between successive R-waves in an electrocardiogram [24, 25] in the following way:

1 $$\begin{aligned} \mathbf {RR}= (RR_{1}, RR_{2}, \ldots , RR_{N}). \end{aligned}$$

Let us also define a derivative, summed and mean-subtracted time series:

2 $$\begin{aligned} y(k) = \sum \limits _{i=1}^{k}RR_i-\overline{RR}, \end{aligned}$$

where $\overline{RR}$ stands for the mean of the whole time series and *y*(*k*) defines the so-called box of length *n* within which a trend is found and subtracted. Since our aim is to identify rising and falling trends we only use first order (linear) polynomials

3 $$\begin{aligned} y(k) = y_{n}(k) + \epsilon _k, \end{aligned}$$

where $y_n(k)$ is a line fit with slope $a_n$ and intercept $b_n$ to the specific box and $\epsilon _k$ is the error term we are interested in studying. A mean-square root function is defined as:

4 $$\begin{aligned} F(n)= \sqrt{\frac{1}{N}\sum \limits _{k=1}^{N}\left( y(k)-y_n(k)\right) ^2}, \end{aligned}$$

and calculated over all the scales which are available in the studied time series — in practice *n* changes from 4 to *N*/4, where *N* is the length of the time series. The values of $\log _{10}(F(n))$ are plotted against $\log _{10}(n)$ and if the resulting plot follows a straight line, the existence of power law is concluded in the scaling of the mean-square root function, i.e.,

5 $$\begin{aligned} F(n)\sim n^{\alpha }, \end{aligned}$$

where $\alpha$ is the scaling exponent. The values of this exponent are interpreted as signifying the presence of negative long-range correlations ($0<\alpha <0.5$), white noise ($\alpha = 0.5$), positive long-range correlation ($0.5<\alpha <1$), 1/*f* noise ($\alpha = 1$), long-range correlations not following the power law ($1<\alpha <1.5$) or the consistency of the detrended time series with random walk ($\alpha = 1.5$) [21–23].

The fact that local trends in the boxes can be linear makes it possible to define two different mean-square root functions, depending on the sign of the tangent of the fitted line.

Defining *M* as the overall number of segments and $M_n^{+}$ as the number of segments in which the trend is increasing (or, using Eq. (3), $a_n>0$) we define:

6 $$F^+(n)=\sqrt{\frac1{M_n^+n}\sum_{j=1}^M\delta^+(j)\sum_{k=1}^n\left(y\left((j-1)\cdot n+k\right)-y_n\;\left((j-1)\cdot n+k\right)\right)^2},$$

and correspondingly for the decreasing trends:

7 $$F^-(n)=\sqrt{\frac1{M_n^-n}\sum_{j=1}^M\delta^-(j)\sum_{k=1}^n\left(y\left((j-1)\cdot n+k\right)-y_n\;\left((j-1)\cdot n+k\right)\right)^2},$$

where the outer summation (over *j*) goes over all boxes, $\delta (\pm )$ selects boxes with increasing (Eq. (6)) or decreasing trends (Eq. (7)) exclusively (compare [14, 16]).

If the above functions are presented on a doubly logarithmic scale with *n*, two scaling exponents:

8 $$\begin{aligned} F^{\pm }(n)\sim n^{\alpha ^{\pm }}, \end{aligned}$$

may be defined, provided that the dependence is linear.

In [13] the local version of ADFA was developed in which the scaling exponents $\alpha ^{\pm }$ are calculated in a window moving along the analyzed time series.

In [15] we applied the local version of ADFA to the time series of $RR$ intervals (moving window length 100) and the result was that there is a highly statistically significant asymmetric effect with $\alpha ^{+} < \alpha ^{-}$. In [16] we systematically analyzed this effect and found that it was present in both global and local versions of ADFA with the use of windows of length 100 through 1000 in 30-min ECG recordings, but it was much weaker in the global version.

### Variance-based heart rate variability descriptors

The variance of the time series (1) is defined in the following way [1, 24, 26]:

9 $$\begin{aligned} SDNN^{2}=\frac{1}{N}\sum \limits _{i=1}^{N}(RR_{i}-\overline{RR})^{2}, \end{aligned}$$

where *N* is the length of the time series.

Variance $SDNN^{2}$ can be partitioned into short-term and long-term variability in the following way [1, 24, 26, 27]:

10 $$\begin{aligned} SDNN^{2} = \frac{SD1^2 + SD2^2}{2}. \end{aligned}$$

The reasons for calling $SD1^2$ and $SD2^2$ the short-term and long-term variability and the details on their calculations are explained in detail in [1, 24, 26].

### Variance-based heart rate asymmetry descriptors

The source of variance-based HRA descriptors is the decomposition of variance-based HRV descriptors, such as $SDNN^2$, $SD1^{2}$ and $SD2^2$ into parts which only depend on decelerations or accelerations.

In [1] it was shown that $SD1^2$ can be partitioned into two parts dependent separately on decelerations and accelerations in the following way:

11 $$\begin{aligned} SD1^{2}=SD1_{d}^{2}+SD1_{a}^{2}. \end{aligned}$$

In [17] it was demonstrated that long-term variability may be partitioned in the following way:

12 $$\begin{aligned} SD2^{2}=SD2_{d}^{2}+SD2_{a}^{2}, \end{aligned}$$

and the full variance may be partitioned in the following way:

13 $$\begin{aligned} SDNN^{2}=SDNN_{d}^{2}+SDNN_{a}^{2}. \end{aligned}$$

For numerical and algorithmic details of the above see [1, 17].

The respective parts of variance can be normalized in order to minimize interpersonal variability [1, 17]. For short-term variance:

14 $$\begin{aligned} C1_{d}=\frac{SD1_{d}^{2}}{SD1^{2}}, \qquad C1_{a}=\frac{SD1_{a}^{2}}{SD1^{2}}, \end{aligned}$$

and there is:

15 $$\begin{aligned} C1_{d}+C1_{a}=1. \end{aligned}$$

For long-term variance:

16 $$\begin{aligned} C2_{d}=\frac{SD2_{d}^{2}}{SD2^{2}}, \qquad C2_{a}=\frac{SD2_{a}^{2}}{SD2^{2}}, \end{aligned}$$

where:

17 $$\begin{aligned} C2_{d}+C2_{a}=1. \end{aligned}$$

And finally, for total variance;

18 $$\begin{aligned} C_{d}+C_{a}=1, \end{aligned}$$

where:

19 $$\begin{aligned} C_{d}= \frac{SDNN_{d}^{2}}{SDNN^{2}},\qquad C_{a}= \frac{SDNN_{a}^{2}}{SDNN^{2}}. \end{aligned}$$

The above descriptors, when applied to the $RR$ intervals time series, reveal a strong asymmetry of this object. First of all, the contribution of heart rate decelerations to short-term variability is greater than that of accelerations, i.e., $SD1_d^2 > SD1_a^2$. In long-term variability this is reversed with $SD2_d^2<SD2_a^{2}$, and this is also true in the case of total variability with $SDNN_{d}^{2}<SDNN_{a}^2$. If the normalized contributions defined above (14), (16), (19) are taken into account, the asymmetry in short-term, long-term and total variability may be respectively expressed as $C1_d>0.5$, $C2_d<0.5$ and $C_d<0.5$.

### The runs method

**Fig. 1:**
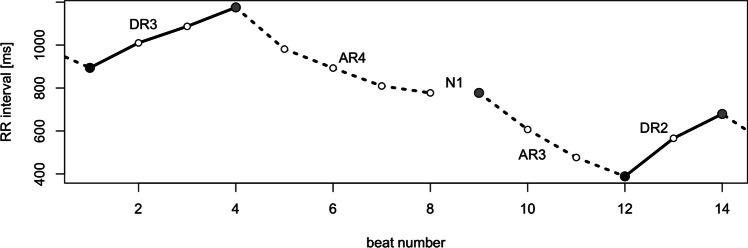
An example of the partitioning of an $RR$ intervals time series into monotonic runs. The runs are marked as DRn (Deceleration Run of length n) and ARn (Acceleration run of length n); the Nn symbols stand for neutral runs which may break the deceleration/acceleration runs. Full black circles denote beginnings of decelerations runs and full gray circles mark the beginnings of accelerations runs — these can be thought of as reference points for the respective runs

A *run* is an uninterrupted sequence of $RR$ intervals which constantly shortens (heart rate accelerates) or constantly lengthens (heart rate decelerates) or constantly does not change, and which is preceded and followed by a different type of run. Figure 1 shows the partitioning of a segment of an $RR$ intervals time series into disjoint accelerating and decelerating runs. It can be easily noted that this partitioning is unambiguous [2]. A detailed definition of runs may be found in [2].

### Runs-based descriptors

#### Number of runs

The most natural descriptor is the number of runs of a specific type. So, by $DR_i$ we will denote the number of deceleration runs of length *i*, and $AR_i$ will mean the number of acceleration runs of length *i*.

#### Entropic descriptors of runs of decelerations and accelerations

In [2] the Shannon entropy [28] associated with the distribution of decelerating and accelerating runs was partitioned into parts depending only on decelerations or only on accelerations in the following way (dropping the device-dependent neutral runs):

$$\begin{aligned} H_{R}=H_{DR} + H_{AR}. \end{aligned}$$

The mathematical details of the above formulas may be found in [2].

In [2, 18] it was found that the runs of accelerations are longer in terms of the number of beats than the runs of decelerations. In [2] it was found that $H_{DR}<H_{AR}$. Both these effects are highly statistically significant.

The runs method turned out to have a significant predictive value for long-term survival in patients after myocardial infarction [18] and in patients who underwent clinically indicated exercise test [19].

Runs have also been found to be very useful in studying and predicting sleep apnea [20, 29] as well as selecting patients who will respond to the proper treatment of obstructive sleep apnea. It has also been applied to the diagnosis of late sepsis in neonates [30].

## Methods

We used 388 stationary 30-min ECG recordings from healthy young subjects, age range 20–40 years, 233 women. The study was performed at rest in the supine position, and the subjects were kept quiet in a neutral environment. The subjects were allowed to breathe spontaneously during the whole study. The 30-min recording was taken after a preceding 15-min period used for cardiovascular adaptation. The ECG curves were sampled at 1600 Hz with the use of the analog-digital converter (Porti 5, TMSI, Holland). The libRASCH/RASCHlab (v. 0.6.1, www.librash.org, Raphael Schneider, Germany) [31] software was used for post-processing and automatic classification into beats of sinus, ventricular and supraventricular origin as well as artifacts. The automatic classification was reviewed by a trained technician who corrected any wrong classifications of the beats. To obtain the asymmetric descriptors from the annotated $RR$ intervals time series we used in-house, free GPL3 software written in Python, HRAexplorer, which can be reviewed and downloaded at https://github.com/jaropis/HRAExplorer. An interactive online version of this software in the R programming language may be found at https://hraexplorer.com/. The $RR$ intervals time series were carefully filtered for each technique — the specifics of dealing with ectopic beats are described in [25] and [2]. The above-mentioned software uses all these filtering techniques.

Since both theory and the Shapiro-Wilk test reject the normality of all HRA descriptors, the non-parametric paired Wilcoxon test was used to establish asymmetric relations. The binomial test was used to establish the departure from 0.5 of recordings exhibiting HRA, which would be the case in symmetric (e.g., shuffled) data.

In the present paper we used the local version of ADFA with jumping window of length 100 beats, since in a previous study [16] it was the shortest window in which asymmetry was clear and consistent with longer windows. By *jumping* windows we mean disjoint windows of length 100 fully covering the analyzed recording. This can be contrasted with a *sliding* window which means a window sliding along the recording, moving by either one beat or by a time unit — for details see [16, 32]. To calculate $\alpha ^{+}$ and $\alpha ^{-}$ in-house, free, GPL3 software written in Python with the use of Cython (https://github.com/kosmo76/adfa) was used. Any ectopic beats were linearly interpolated according to [33].

The time series of $\alpha ^{+}$ and $\alpha ^{-}$ obtained for each recording were summarized by medians for the purpose of comparisons. Since the mean values of $\alpha ^{+}$ and $\alpha ^{-}$ did not have normal distribution (Shapiro-Wilk test), the non-parametric paired Wilcoxon test was used for their comparison.

The association analyses between ADFA asymmetric exponents and the descriptors of HRA were carried out with the use of the non-parametric Spearman correlation test. To check whether HRA entails $\alpha ^{+}<\alpha ^{-}$ we built the univariate logistic regression models for $2\times 2$ contingency tables tabulating the existence of HRA and $\alpha ^{+}<\alpha ^{-}$.

All statistical calculations were carried out with the use of the R statistical language and its libraries.

## Results

### Presence of asymmetry in the studied signals

#### Presence of asymmetry in ADFA

The median values of the scaling exponents were $\alpha ^{+}=0.951$ (IQR (0.825, 1.052)), $\alpha ^{-}=0.993$ (IQR (0.885, 1.099)), the *p* value is $< 0.0001$. These results should be analyzed in view of the possible values that $\alpha ^{\pm }$ may take (see discussion after formula (5)). If the median values above were different by a larger amount, this would mean a total flip in the properties of the noise after detrending.

The $\alpha ^{+}<\alpha ^{-}$ was present in 278 cases, which is 74% of the entire group, the binomial test for this value to be consistent with 50% gives the *p*-value < 0.0001

#### Presence of asymmetry in the variance-based descriptors

The presence of asymmetry in the variance-based descriptors was established by comparing the deceleration- and acceleration-based parameters as well as checking whether globally the proportion of subjects with a specific type of asymmetry was different from the theoretically expected value of 0.5 if there is no asymmetry [1, 17].

##### Short-term asymmetry

The parameters of the distribution of $SD1_d$, $SD1_a$ and $C1_d$ may be found in Table 1. The number of cases in which short-term asymmetry can be established is 291, which is 75% of the entire group, the binomial test for this value to be consistent with 50% gives the *p*-value < 0.0001.

**Table 1:** The distributions of short-term HRA descriptors, i.e., $SD1_d$, $SD1_a$ and $C1_d$ in the studied recordings

	Min.	1st Q.	Median	Mean	3rd Q.	Max.
$SD1_d$	2.704	14.29	20.11	25.11	31.24	140.5
$SD1_a$	2.741	14.22	19.36	22.44	27.67	137.0
$C1_d$	0.3060	0.5002	0.5304	0.539	0.5733	0.7021

##### Long-term asymmetry

The parameters of the distribution of $SD2_d$, $SD2_a$ and $C2_d$ may be found in Table 2. Long-term asymmetry is present in 269, which is 69.3% of the entire group, the binomial test for this value to be consistent with 50% gives the *p*-value < 0.0001.

**Table 2:** The distributions of long-term HRA descriptors, i.e., $SD2_d$, $SD2_a$ and $C2_d$ in the studied recordings

	Min.	1st Q.	Median	Mean	3rd Q.	Max.
$SD2_d$	17.82	41.11	52.74	55.00	66.16	138.7
$SD2_a$	17.54	41.56	55.18	59.17	71.35	155.4
$C2_d$	0.3179	0.4508	0.4827	0.474	0.5042	0.5662

##### Total asymmetry

The parameters of the distribution of $SDNN_d$, $SDNN_a$ and $C_d$ may be found in Table 3.

**Table 3:** The distributions of total HRA descriptors, i.e., $SDNN_d$, $SDNN_a$ and $C_d$ in the studied recordings

	Min.	1st Q.	Median	Mean	3rd Q.	Max.
$SDNN_d$	12.75	31.29	40.61	43.16	51.77	139.6
$SDNN_a$	12.55	31.34	41.64	44.97	54.41	146.5
$C_d$	0.3670	0.4698	0.4902	0.4855	0.5035	0.5604

##### Presence of asymmetry in the runs based descriptors

**Fig. 2:**
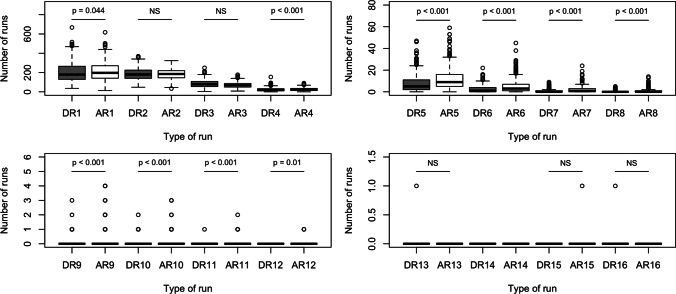
Distributions of monotonic runs along with the comparisons with the paired Wilcoxon test

The summary of the acceleration and deceleration runs distributions may be found in Fig. 2 and in Table 4.

It can be concluded that the asymmetric effect, i.e., runs of accelerations being longer than those of decelerations, can be observed for runs of lengths 4 through 12, with lengths 1–3 and over 12 being not statistically significant or too few to carry out the statistical tests. Thus, the runs-based descriptors demonstrate the presence of HRA in the studied group.

**Table 4:** The distributions of monotonic runs in the studied recordings

RL	DR	IQR DR	# DR	AR	IQR AR	# AR	p
1	179.0	(129.75–265)	388	196.5	(129.75–265)	388	p = 0.044
2	180.0	(140–225)	388	185.0	(140–225)	388	NS
3	79.0	(54.75–108)	388	68.0	(54.75–108)	388	NS
4	17.5	(11–31.5)	386	21.5	(11–31.5)	387	p < 0.001
5	5.0	(2–11)	363	9.0	(2–11)	377	p < 0.001
6	1.0	(0–4)	279	3.0	(0–4)	341	p < 0.001
7	0.0	(0–1)	148	1.0	(0–1)	257	p < 0.001
8	0.0	(0–0)	68	0.0	(0–0)	151	p < 0.001
9	0.0	(0–0)	22	0.0	(0–0)	71	p < 0.001
10	0.0	(0–0)	9	0.0	(0–0)	35	p < 0.001

The line labels indicate the run length (RL), DR (AR) stands for the number of the deceleration (acceleration) runs of a specific length, # with DR (AR) means the number of recording containing runs of the specified type and length

Table 5 demonstrates the distributions of runs entropy for $H_{DR}$ and $H_{AR}$ shows the comparison between the two types.

The direct comparison between the entropic runs summaries yields $p<0.0001$, so there is a highly statistically significant asymmetric effect.

**Table 5:** The distributions of entropic HRA descriptors, i.e., $H_{DR}$ and $H_{AR}$

	Min.	1st Q.	Median	Mean	3rd Q.	Max.
$H_{DR}$	0.6865	0.9351	1.024	1.023	1.107	1.388
$H_{AR}$	0.7907	1.0100	1.096	1.115	1.214	1.601

### Associations between variance-based HRA descriptors and ADFA

#### Associations between variance-based descriptors and local asymmetric scaling exponents

The associations between variance-based HRA descriptors and ADFA exponents were studied with the use of the Spearman correlation as well as *loess* type regression to visualize the type of association. The results are presented in Table 6.

**Table 6:** Associations between variance-based HRA descriptors and ADFA scaling exponents

Descriptor	$\alpha ^{+}$	$\alpha ^{-}$
*SD*1	−0.798 ($p<0.0001$)	−0.561 ($p<0.0001$)
*SD*2	−0.475 ($p<0.0001$)	−0.198 ($p<0.0001$)
*SDNN*	−0.544 ($p<0.0001$)	−0.269 ($p<0.0001$)
$SD1_d$	−0.784 ($p<0.0001$)	−0.529 ($p<0.0001$)
$SD1_a$	−0.809 ($p<0.0001$)	−0.6 ($p<0.0001$)
$C1_d$	−0.277 ($p<0.0001$)	0.086 (NS)
$SD2_d$	−0.454 ($p<0.0001$)	−0.201 ($p<0.0001$)
$SD2_a$	−0.487 ($p<0.0001$)	−0.195 ($p<0.0001$)
$C2_d$	0.417 ($p<0.0001$)	0.077 (NS)
$SDNN_d$	−0.54 ($p<0.0001$)	−0.279 ($p<0.0001$)
$SDNN_a$	−0.546 ($p<0.0001$)	−0.26 ($p<0.0001$)
$C_d$	0.335 ($p<0.0001$)	0.02 (NS)

From the table above it can be seen that the asymmetric scaling exponents are significantly associated with both HRV and HRA magnitudes. The associations between asymmetric contributions of HRA to short-term, long-term and total variability and the asymmetric exponents are significant for decelerations and not significant for accelerations. Therefore it is necessary to study this in more detail. This is undertaken in the next section.

#### Associations between the presence of asymmetry in variance-based descriptors and the presence of asymmetry in local asymmetric scaling exponents

To answer the question whether or not the asymmetry observable in variance-based descriptors is related to the asymmetry observable in the scaling exponents is actually present rather than there being just an association between $\alpha ^{\pm }$ and the variance of time series we first build a $2\times 2$ contingency table between the two categorical variables. Then for each type of asymmetry we build a logistic regression model to study the strength and significance of the association, i.e., the predictor in each case is the presence of asymmetry expressed according to the standard definition, e.g., using the inequalities from points 1.3, 1.4 and 1.5. The presence of asymmetry is coded as 1.

The relations between various types of HRA and ADFA are summarized in Table 7. The results of the logistic model of the above asymmetry as predictor of $\alpha ^{+}<\alpha ^{-}$ are presented in Table 8.

**Table 7:** Associations between occurrence of HRA and the asymmetry in ADFA

	$\alpha ^{+} > \alpha ^{-}$	$\alpha ^{+} < \alpha ^{-}$		$\alpha ^{+} > \alpha ^{-}$	$\alpha ^{+} < \alpha ^{-}$		$\alpha ^{+} > \alpha ^{-}$	$\alpha ^{+} < \alpha ^{-}$
$C1_d < 0.5$	56	41	$C2_d > 0.5$	68	51	$C_d > 0.5$	68	55
$C1_d > 0.5$	54	237	$C2_d < 0.5$	42	227	$C_d < 0.5$	42	223

**Table 8:** Logistic regression analysis results for the associations between HRA and the presence of ADFA asymmetry

Asymmetry type	Coeff.
$C1_d > 0.5$	1.791 ($p<0.0001$)
$C2_d < 0.5$	1.975 ($p<0.0001$)
$C_d < 0.5$	1.882 ($p<0.0001$)

It can be concluded that variance-based heart rate asymmetry and asymmetry in local ADFA are related.

### Associations between runs-based HRA descriptors and ADFA

Tables 9 and 10 demonstrate the correlations between deceleration / acceleration runs and ADFA asymmetric exponents. The associations are quite strong for runs of length greater than 3. This may mean that there is both an association between heart rate asymmetry and ADFA and an association between the presence of various length runs and ADFA. Thus it is necessary to study the co-occurrence of both types of asymmetry.

**Table 9:** Associations between deceleration runs and the ADFA scaling exponents

	$\alpha ^{+}$	$\alpha ^{-}$
DR1	0.061 (NS)	0.064 (NS)
DR2	−0.325 ($p<0.0001$)	−0.42 ($p<0.0001$)
DR3	0.188 (2e-04)	0.076 (NS)
DR4	0.478 ($p<0.0001$)	0.492 ($p<0.0001$)
DR5	0.645 ($p<0.0001$)	0.681 ($p<0.0001$)
DR6	0.684 ($p<0.0001$)	0.643 ($p<0.0001$)
DR7	0.574 ($p<0.0001$)	0.561 ($p<0.0001$)
DR8	0.524 ($p<0.0001$)	0.464 ($p<0.0001$)
DR9	0.257 ($p<0.0001$)	0.216 ($p<0.0001$)
DR10	0.231 ($p<0.0001$)	0.239 ($p<0.0001$)

**Table 10:** Associations between acceleration runs and the ADFA scaling exponents

	$\alpha ^{+}$	$\alpha ^{-}$
AR1	0.091 (NS)	−0.011 (NS)
AR2	−0.245 ($p<0.0001$)	−0.43 ($p<0.0001$)
AR3	0.091 (NS)	0.109 (0.0321)
AR4	0.211 ($p<0.0001$)	0.41 ($p<0.0001$)
AR5	0.267 ($p<0.0001$)	0.52 ($p<0.0001$)
AR6	0.345 ($p<0.0001$)	0.585 ($p<0.0001$)
AR7	0.396 ($p<0.0001$)	0.581 ($p<0.0001$)
AR8	0.364 ($p<0.0001$)	0.513 ($p<0.0001$)
AR9	0.279 ($p<0.0001$)	0.427 ($p<0.0001$)
AR10	0.266 ($p<0.0001$)	0.318 ($p<0.0001$)

#### Associations between the presence of asymmetry in runs-based descriptors and the presence of asymmetry in local asymmetric scaling exponents

We use the same method as above for runs of length greater than 3, since for these runs the heart rate asymmetry is clearly observable. The results of the logistic regression models for all the analyzed run lengths can be found in Table 12.

**Table 11:** Associations between occurrence of HRA in runs of length 4–10 and the asymmetry in ADFA

	$\alpha ^{+} > \alpha ^{-}$	$\alpha ^{+} < \alpha ^{-}$		$\alpha ^{+} > \alpha ^{-}$	$\alpha ^{+} < \alpha ^{-}$
DR4 > AR4	73	93	DR5 > AR5	57	67
DR4 < AR4	37	185	DR5 < AR5	53	210
DR6 > AR6	52	64	DR7 > AR7	38	36
DR6 < AR6	40	204	DR7<AR7	38	171
DR8> AR8	23	24	DR9>AR9	10	9
DR8< AR8	24	101	DR9<AR9	11	58
DR10 > AR10	2	5			
DR10< AR10	7	26

**Table 12:** Logistic regression analysis results for the associations between the presence of ADFA asymmetry and runs asymmetry

Runs asymmetry	Coeff.
DR4< AR4	1.367 ($p<0.0001$)
DR5< AR5	1.215 ($p<0.0001$)
DR6< AR6	1.422 ($p<0.0001$)
DR7< AR7	1.558 ($p<0.0001$)
DR8< AR8	1.395 ($p<0.0001$)
DR9< AR9	1.768 ($p<0.0001$)
DR10< AR10	0.396 ($NS$)

From both Tables 11 and 12 it can be concluded that the presence of asymmetry in monotonic runs predicts the presence of asymmetry in ADFA.

#### The association between asymmetry in the entropic HRA descriptors and ADFA

The associations between $\alpha ^{\pm }$ and the quantities summarizing all the above runs, i.e., $H_{DR}$ and $H_{AR}$ are presented in Table 13.

**Table 13:** Associations between $H_{DR}$ and $H_{AR}$ and the asymmetry in ADFA

	$\alpha ^{+}$	$\alpha ^{-}$
$H_{DR}$	0.756 ($p<0.0001$)	0.68 ($p<0.0001$)
$H_{AR}$	0.319 ($p<0.0001$)	0.601 ($p<0.0001$)

As we did for the other descriptors, let us build the $2\times 2$ contingency table (Table 14) and the logistic model for the co-occurrence of the two types of asymmetry.

**Table 14:** Associations between occurrence of HRA in the entropic descriptors and the asymmetry in ADFA

	$\alpha ^{+} > \alpha ^{-}$	$\alpha ^{+} < \alpha ^{-}$
$H_{DR} > H_{AR}$	57	40
$H_{DR} < H_{AR}$	53	238

The coefficient of the model is 1.856 with $p<0.0001$, so again the association is strong.

### HRA as a predictor for the occurrence of asymmetry in ADFA

If HRA occurrence is treated as a predictor of ADFA then the positive predictive values for the variance-based asymmetry are 0.85, 0.82 and 0.8 with sensitivities 0.81, 0.84 and 0.84 for short-term, long-term and total HRA, 0.81 with sensitivity 0.85 for $H_{AR}<H_{DR}$ and 0.67, 0.76, 0.76, 0.83, 0.81, 0.87 and 0.84 with sensitivities 0.82, 0.83, 0.8, 0.84, 0.82, 0.81, 0.84 and 0.79 for HRA in runs of length 4 through 10.

Clearly, in all the types of descriptors, the presence of asymmetry in HRA entails the presence of asymmetry in ADFA.

## Discussion

In the present paper we have shown that HRA in both its versions presented here, i.e., variance based and runs based, is associated with the asymmetry observable in ADFA, i.e., $\alpha ^{+}<\alpha ^{-}$. The study was carried out in a group of healthy young people from whom stationary 30-min-long recordings were obtained.

In the studied group we observed clear and highly statistically significant heart rate asymmetry at all time scales, i.e., short-term ($C1d>0.5$), long-term ($C2d<0.5$) and total ($Cd<0.5$).

Runs-based asymmetry is also clearly visible and significant for all observable runs of length $> 3$ as well as in the summary runs-based descriptors, i.e., $H_{DR}<H_{AR}$.

This group also exhibits a clear and highly statistically significant ADFA asymmetry, defined as $\alpha ^{+} < \alpha ^{-}$.

We have found that both scaling exponents are highly statistically significantly associated with the heart rate variability of the analyzed recordings, i.e., they were associated with total ($SDNN^{2}$), long-term ($SD2^{2}$) and short-term ($SD1^{2}$) variability. For this reason they were also associated with the unnormalized HRA Poincaré plot descriptors like $SD1_{d/a}^2$, $SD2_{d/a}^2$ and $SDNN_{d/a}^{2}$. The associations between the scaling exponents and the *magnitude* of the relative contributions to asymmetry ($C1_d$, $C2_d$, $C_d$) were significant for $\alpha ^{+}$, but not significant for $\alpha ^{-}$. At this point it is probably impossible to interpret this result.

The associations of $\alpha ^{\pm }$ with the *magnitudes* of the runs-based HRA descriptors are also highly statistically significant.

However, the strongest associations between ADFA and HRA in the $RR$ intervals time series are observable in the agreement in between the two types. From the numbers presented in Section 3.4 on the predictive power of HRA for ADFA asymmetry, it is almost certain that both approaches describe the same physiological phenomenon, even though they use totally different methods, assumptions and even different language (HRA is based on statistical considerations, and ADFA on studying long-range correlations in dynamic systems).

An important limitation of this study should be raised at this point. Since the recordings used in the present study are quite short, the $\alpha ^{\pm }$ cannot be considered measures of fractality and they will be strongly related with the variance of the recording. Therefore, the results involving variance-based HRA descriptors are weakened by this observation. However, the relation with the normalized descriptors is much more robust to this effect since the dependence on variance is largely eliminated by normalizing by variance-based parameters. Additionally, the results relating the incidence of asymmetry in ADFA with HRA should be fully resistant to this effect.

The explanation of HRA has not yet been established. In the crudest approximation of the Autonomic Nervous System (ANS) it can be said that the parasympathetic branch of the ANS is responsible for decelerations and the sympathetic branch is responsible for accelerations. In this picture it might be tempting to ascribe the deceleration-based descriptors ($SD1_d$, $SD2_d$, $SDNN_d$, $C1_d$, $C2_d$, $C_d$, DRx as well as $\alpha ^{+}$) to the parasympathetic branch and the rest to the sympathetic branch. Yet, in reality, both branches can lead to both accelerations and decelerations through activation or deactivation. It would be more prudent to state that HRA reflects the interaction between both branches as well as describing the patterns of accelerations and decelerations. $\alpha ^{\pm }$ in the approach assumed in ADFA are influenced by both accelerations and decelerations and reflect the differences in noise left over after removing *linear* trends. Thus, their asymmetric behavior most possibly reflects the different interactions, both long and short term, present during decelerating and accelerating *trends*.

Possibly the best way in which the two types of asymmetry can be related is through the monotonic runs. It is fair to hypothesize that, since falling and rising trends consist of individual runs, rising trends in the $RR$ intervals time series should be dominated by decelerating runs and falling trends by accelerating runs. Since, as we have shown in this and other papers, the dynamics of these runs differ, the long-range correlations reflected by the ADFA scaling exponents, should also differ.

The above hypothesis is strictly mathematical. As far as physiological relation is concerned,we can safely hypothesize that both measures are linked through a common, underlying physiological process. One such process which immediately comes to mind is respiratory sinus arryhythmia, which is a strong driver of heart rate variability. However, as demonstrated in [34], the link between HRA and RSA is by no means clear. The authors find no link between period variability asymmetry and respiratory sinus arrhythmia in healthy and chronic heart failure individuals. This means that further, physiologically oriented studies are necessary.

Since this is a strictly observational study we refrain from physiological explanation for HRA and its relation with ADFA as this would call for a full paper, we would, however, like to note that a few interesting and convincing physiological mechanisms have been identified in [35]. Another point that should be raised at this point is that we use a specific approach to HRA, namely the variance-based and runs-based descriptors. There are other approaches, like using predictability markers [36] which could also shed light on the analyzed problem.

To sum up, ADFA asymmetry is associated with HRA and one is an almost perfect predictor of the other.

## Conclusion

ADFA, which is a new method for studying asymmetry in time series has been applied to the time series of $RR$ intervals. It has been found that it is associated to other approaches to asymmetry in this time series. Since ADFA has unique properties, like the ability to study long-range correlations or scaling behavior of the time series, it is a promising direction in the HRA analysis, even though it does have some interpretational difficulties.
